# Exploring the perspectives of primary care providers on use of the electronic Patient Reported Outcomes tool to support goal-oriented care: a qualitative study

**DOI:** 10.1186/s12911-021-01734-0

**Published:** 2021-12-29

**Authors:** Hardeep Singh, Farah Tahsin, Jason Xin Nie, Brian McKinstry, Kednapa Thavorn, Ross Upshur, Sarah Harvey, Walter P. Wodchis, Carolyn Steele Gray

**Affiliations:** 1 Bridgepoint Collaboratory for Research and Innovation, Lunenfeld-Tanenbaum Research Institute, Sinai Health, Toronto, ON Canada; 2 Department of Occupational Science and Occupational Therapy, Temerty Faculty of Medicine, University of Toronto, 500 University Avenue, Toronto, Canada; 3 March of Dimes Canada, Toronto, Canada; 4 Institute of Health Policy, Management and Evaluation, Dalla Lana School of Public Health, University of Toronto, Toronto, ON M5T 3M6 Canada; 5 Institute for Better Health, Trillium Health Partners, Mississauga, ON L5B 1B8 Canada; 6 Usher Institute, University of Edinburgh, Edinburgh, EH16 4UX UK; 7 Ottawa Hospital Research Institute, Ottawa, ON K1Y 4E9 Canada; 8 School of Epidemiology and Public Health, University of Ottawa, Ottawa, ON K1N 6N5 Canada; 9 Department of Family and Community Medicine, Temerty Faculty of Medicine, University of Toronto, Toronto, Canada; 10 Logibec Inc., 1751, Richardson Street, Suite 1.060, Montréal, QC H3K 1G6 Canada

**Keywords:** Implementation science, Primary health care, Digital technology

## Abstract

**Background:**

Digital health technologies can support primary care delivery, but clinical uptake in primary care is limited. This study explores enablers and barriers experienced by primary care providers when adopting new digital health technologies, using the example of the electronic Patient Reported Outcome (ePRO) tool; a mobile application and web portal designed to support goal-oriented care. To better understand implementation drivers and barriers primary care providers’ usage behaviours are compared to their perspectives on ePRO utility and fit to support care for patients with complex care needs.

**Methods:**

This qualitative sub-analysis was part of a larger trial evaluating the use of the ePRO tool in primary care. Qualitative interviews were conducted with providers at the midpoint (i.e. 4.5–6 months after ePRO implementation) and end-point (i.e. 9–12 months after ePRO implementation) of the trial. Interviews explored providers’ experiences and perceptions of integrating the tool within their clinical practice. Interview data were analyzed using a hybrid thematic analysis and guided by the Technology Acceptance Model. Data from thirteen providers from three distinct primary care sites were included in the presented study.

**Results:**

Three core themes were identified: (1) *Perceived usefulness*: perceptions of the tool’s alignment with providers’ typical approach to care, impact and value and fit with existing workflows influenced providers’ intention to use the tool and usage behaviour; (2) *Behavioural intention*: providers had a high or low behavioural intention, and for some, it changed over time; and (3) *Improving usage behaviour*: enabling external factors and enhancing the tool’s perceived ease of use may improve usage behaviour.

**Conclusions:**

Multiple refinements/iterations of the ePRO tool (e.g. enhancing the tool’s alignment with provider workflows and functions) may be needed to enhance providers’ usage behaviour, perceived usefulness and behavioural intention. Enabling external factors, such as organizational and IT support, are also necessary to increase providers’ usage behaviour. Lessons from this study advance knowledge of technology implementation in primary care.

***Trial registration*:**

Clinicaltrials.gov Identified NCT02917954. Registered September 2016, https://www.clinicaltrials.gov/ct2/show/study/NCT02917954

**Supplementary Information:**

The online version contains supplementary material available at 10.1186/s12911-021-01734-0.

## Background

Primary care providers estimate that about a quarter of patients for whom they provide direct care are “complex” [1], and this number is anticipated to grow as the world’s population ages [2]. Care provided to patients with complex care needs is complicated, requiring management of multiple competing health demands and side effects of multiple medications [3–6], as well as managing non-medical barriers to care, such as mental health and socio-economic challenges [1, 3]. Thus, primary care visits “are more packed” as providers must address multiple medical issues per visit [3, 7]. Delivering high quality care to patients with chronic conditions is challenging as it requires three times the amount of time that patients are typically allotted [3, 8].

Digital health solutions may help primary care better address the needs of patients with chronic conditions through improved access to communication tools, symptom monitoring and self-management support [9, 10]. For instance, mobile health (mHealth) can allow providers to monitor patients’ symptoms, medication adherence and activity patterns outside of a primary care clinic [9]. Also, patient portals and electronic health records can enhance communication of health information (e.g. care plans) between providers and between providers and patients/caregivers [9]. Despite the potential and effectiveness of health technologies [11, 12], the rate of technology adoption by healthcare providers remains low [13]. Barriers to technology adoption can relate to technology use (e.g. a lack of fit with existing workflows, poor usability and negative impact on the patient-provider relationship) and the technology implementation challenges (e.g. lack of technical support) [9, 11, 13–16]. Other factors influencing technology adoption are provider buy-in and the perceived utility or value of a tool [12, 13]. While numerous barriers and facilitators to the adoption of technology in primary care have been previously reported, there is a remaining gap in understanding the “how and why” of these factors using exploratory approaches [17]. Primary care is unique given variable team compositions, organizational structures, cultures and work processes across different primary care settings [17]. In addition, the research and implementation culture poses significant challenges to testing, evaluating and implementing novel research approaches in primary care [17, 19].

This study addresses knowledge gaps related to technology implementation in primary care by analyzing provider experiences using the electronic Patient Reported Outcomes (ePRO) within three distinct clinical environments. As the number of providers who deliver person-centered care through multiple different technologies is increasing [20–22], the findings of this study can be transferable to those patient-facing devices that promote person-centered care for patients with chronic conditions. The ePRO tool is a patient mHealth app and provider web portal that supports the creation and monitoring of goal-oriented plans for older adults with complex care needs in primary care [18, 23, 24]. This study aimed to explore provider perspectives regarding the ePRO’s utility and fit to support care for patients with complex care needs, usage of the ePRO, and considerations to enhance the ePRO’s implementation in primary care settings. Through the application of the Technology Acceptance Model (TAM) [25, 26], commonly used to understand providers’ technology adoption [27], this study generates insights that can inform digital health implementation efforts in primary care.

## Methods

### Design

The qualitative study presented in this paper was part of a larger 15-month pragmatic step-wedge trial, in which the usability and effectiveness of the ePRO tool were evaluated [23, 28, 29]. The stepped-wedge design entailed randomizing sites (n = 6) into long (12 months) or short (9 months) intervention groups, with a sub-set identified as case sites (n = 3) where we collected ethnographic qualitative data alongside trial outcome data. The sub-study presented in this paper is guided by a qualitative interpretive descriptive approach [30, 31], where we analyze qualitative data collected at the three case sites. Given that interpretive description is a method that enables exploration of clinical phenomenon and generation of findings that are suitable for practice use in clinical practice, it was well-suited to address the current study aims [30, 31]. Ethics approval was received from the Research Ethics Board of the University of Toronto and Sinai Health System. All participants provided informed consent before data collection, and all methods complied with relevant guidelines and regulations.

### Research framework

TAM is a framework used to understand and describe users’ intention to use technology [25, 26]. TAM posits that perceived ease of use (PEOU), perceived usefulness (PU) and external factors influence intention to use and actual use (i.e. usage behaviour (UB)) of a technology (see Table 1 for the TAM variables) [25, 32]. This study used the TAM to gain detailed insights into how these multiple factors influence the constructs of BI and UB. The identified influencing factors can be used to refine the ePRO further to enhance BI and UB.

**Table 1:** The technology acceptance model (TAM) domains

TAM domain	Description [26, 32, 37]
Perceived ease of use (PEOU)	The degree to which the provider believes the ePRO is free of effort
Perceived usefulness (PU)	The degree to which the provider believes that using the ePRO enhances their job performance
Behavioural intention (BI)	The strength of a provider’s intention to use the ePRO
Usage behaviour (UB)	Actual use of the ePRO

### Intervention: ePRO tool

As previously described by Steele Gray & colleagues [18, 23, 24], the ePRO tool was designed to support self-management and shared decision making through collaborative use by a patient, caregiver(s) and health care provider(s). To improve adoption, it was co-designed with end-users, including health care providers, content experts, patients with complex care needs and their caregivers [10, 18, 24]. Interviews with six primary care providers during a six-week pilot usability evaluation of an earlier version of the ePRO tool revealed implementation barriers that prevented the adoption of the tool in clinical practice. These included liability concerns related to remote monitoring, lack of interoperability with existing workflows and concerns about time demands to use the tool [18]. Based on the results of the pilot trial, the tool was modified to improve usability prior to the current trial.

### Setting and participants

A purposeful sampling strategy [33] was employed to recruit Family Health Teams (FHTs) to this implementation trial. FHTs were developed to provide comprehensive primary care services to patients through a multidisciplinary care team (e.g., physicians, nurses and allied health providers). The FHTs operated within a single-payer healthcare system in Ontario, Canada [34]. FHTs were recruited using various strategies, including emailing study details to the Association of Family Health Teams of Ontario (AFTHO) and advertising study details at AFHTO meetings and the annual conference [35]. Of the six sites recruited for the trial, providers from three geographically diverse (i.e., urban, rural) sites in Ontario were selected as case sites for deeper qualitative exploration in this study. Initially, four sites agreed to participate and were chosen based on geographic diversity. One site dropped out of the ethnography due to low patient recruitment. Geographic diversity and interest in participating in the ethnography drove case site selection criteria. Of the three sites, one was situated in a rural setting, and two were situated in urban settings [28]. Primary care providers were eligible for this study if they worked at an ePRO trial site and were involved in the care of ≥ one patient (≥ 65 years with ≥ 2 chronic conditions which required frequent care visits) enrolled in the ePRO trial [29].

### Provider training of the ePRO

Providers were requested to use the tool for 9–12 months as part of usual care with patients enrolled in the ePRO trial. Prior to trial initiation, an hour-long workshop, led by the research team, oriented providers to the purpose and functionality of the tool. Providers had practiced using the tool to set, monitor and modify goals with simulated patients collaboratively. Providers also received a training manual and video that detailed the tool’s functions and capabilities and demonstrated the use of the tool. Research coordinators offered additional optional refresher training at the intervention sites at three, six, and nine months after the trial began [29].

### Data collection

Of the 29 providers that were invited, thirteen providers from three primary care sites participated in this study; their characteristics are described in Table 2. Semi-structured interviews (10–60 min) were conducted by phone or in-person by research team members with qualitative expertise (i.e., research coordinators: three female and one male or CSG: female Scientist) at each providers’ workplace. At the beginning of each interview, researchers reminded participants about the purpose of the interview. Interviews were completed at two time-points: midpoint (i.e., ≥ 4.5–6 months of ePRO implementation) and end-point (i.e., ≥ 12-months of ePRO implementation). Field notes were created during and after the interviews. Table 3 contains sample interview questions.

**Table 2:** Participant demographics

Code	Sex	Site #	Discipline	Midpoint interview	Final interview
GBP01	F	Site 1	Nurse	X	X
GBP02	F	Site 1	Nurse	X	X
GBP03	F	Site 1	Dietician	X	X
GBP04	F	Site 1	Nurse	X	X
GBP05	F	Site 1	Nurse Practioner Dietician	X	
GBP06	F	Site 1	Nurse	X	X
MSP01	F	Site 2	Dietician	X	X
MSP02	F	Site 2	Dietician	X	
MSP03	M	Site 2	Physician	X	X
MSP04	F	Site 2	Nurse Practioner Dietician		X
P01OV	F	Site 3	Dietician	X	X
P02OV	M	Site 3	Clinical leader	X	
P03OV	M	Site 3	Nurse		X

**Table 3:** Sample interview questions

Sample interview questions
What has it been like to use ePRO tool in your approach to care? **Probes:** How exactly did you use the tool (e.g. set goals, monitor health outcomes, use the PDF reporting?) In what ways, if any, did having the tool influence your interaction(s) with your patient(s)? How did these interactions change over time as you used the tool? How did your relationship with patients change over time?
Did the ePRO tool adequately capture issues of importance to you as a provider? **Probes:** What do you wish the tool could do that it doesn’t do right now? What is missing or could be added?
How did you find the tool itself? **Probes:** In terms of how you enter information? In terms of how you use the information to help in decision-making?
What were some of the challenges you faced when using the tool? **Probe:** What could be done to help address the challenges you identified?
What organizational supports would be needed to help adopt this tool (e.g. IT support, funding, organizational leadership)? **Probe:** Was there any research team supports you found particularly helpful?

### Data analysis

Interviews were de-identified, audio-recorded and transcribed verbatim. NVivo 11 software (QSR International) and Microsoft Excel (Microsoft Corporation) were used to organize and sort data. Consistent with an interpretive description approach [30, 31], the transcripts were analyzed using a two-stage hybrid inductive and deductive analysis, which allowed us to capture themes not represented in established frameworks [36]. In the first stage, two qualitative researchers (HS: Post-doctoral fellow and occupational therapist and CSG: Scientist) independently read four transcripts and noted segments of data (i.e. inductive codes) considered important to the study objectives [36]. During the analysis, the researchers met to discuss, compare, and refine their inductive codebook. During these meetings, they jointly coded a transcript to ensure their coding schemes aligned and any discrepancies were resolved through discussions. This coding process was repeated four times, after which point they believed their coding schemes aligned and saturation was reached. The transcripts were uploaded onto NVivo 11 software and coded using the final version of the inductive codebook.

In the deductive stage, the data were mapped onto the TAM [26, 37]. Inductive codes were plotted within the variables of the TAM to form the deductive themes. For instance, theme 3 was inductively coded as “recommendations,” but through the TAM deductive analysis, it was mapped into PEOU and external factors. The deductive themes were consolidated through member-checking with participants as well as discussion and agreement between the research team [36].

To enhance rigour within the findings [38, 39], two researchers were involved in the data analysis and met regularly to compare their analytic interpretations (triangulation). An audit trail was produced to document decisions made during analysis [40]. Participant quotes were used to support the researchers’ analytic interpretations [41], and the Standards for Reporting Qualitative Research [42] checklist was followed to improve reporting transparency (see Additional file 1).

## Results

**Fig. 1:**
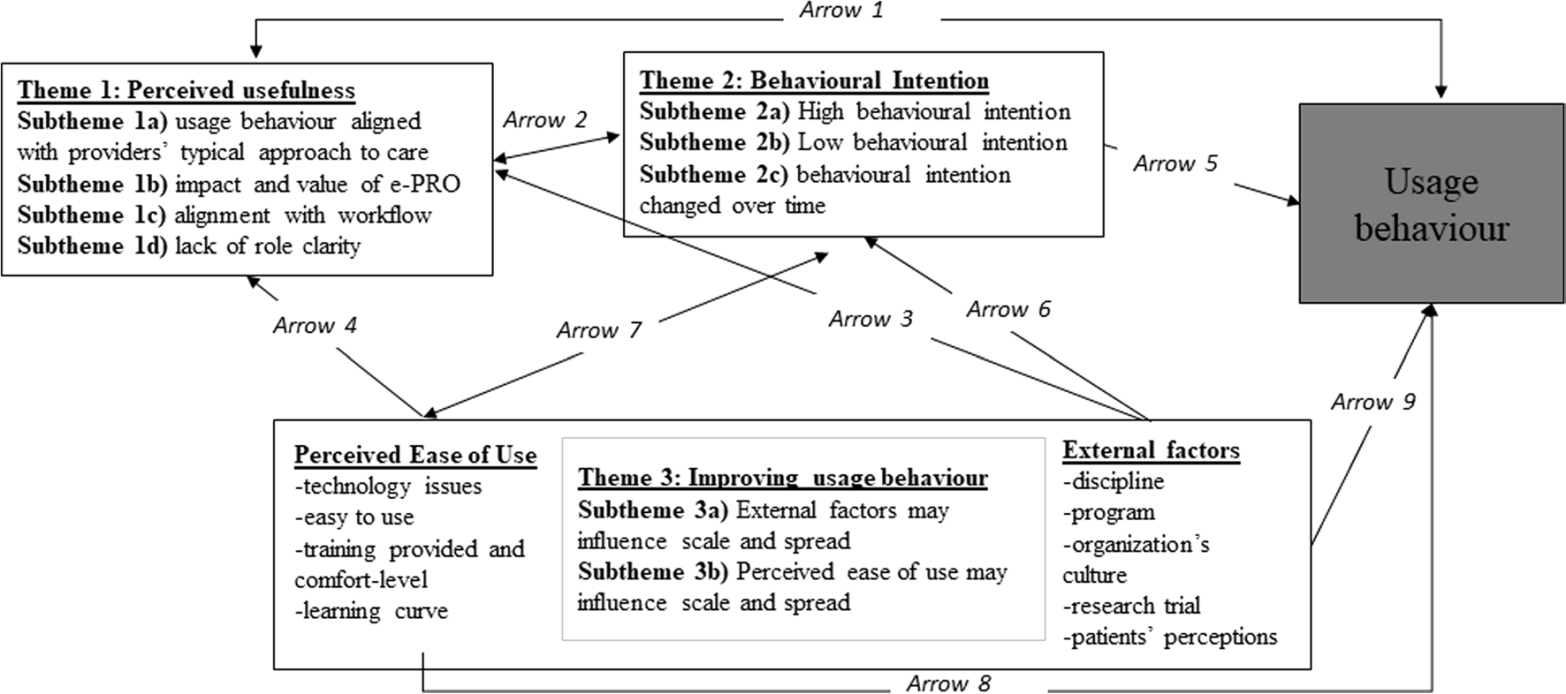
Themes arranged according to categories of the TAM

While UB, as described by providers, indicated that all providers had discontinued use of the ePRO by the final interview, the themes revealed insights about *how* the tool was used. When discussing their UB, providers often referenced themes relating to PU and BI. However, providers focused on PEOU and external supporting factors when recommending how to improve providers’ UB (see Table 4). The findings suggested that *time* was an underlying driver in themes 1 and 3, though not as explicit as it was in theme 2. The following sections are presented according to categories of the TAM. Figure 1 provides an overview of the relationship between themes and subthemes; the arrows represent how one variable within the TAM influenced another and are referenced within the results.

**Table 4:** Summary of the themes, subthemes and supporting quotes

Subthemes	Description	Supporting quotes
*Theme 1: Perceived usefulness*		
Subtheme 1a: Usage behaviour aligned with providers’ typical approach to care	*Varying approach to care and ePRO’s alignment with providers’ care approach*	“Normally, we do goals and then get [patients] into programs. I don’t often see people ongoing…I didn’t even really do a whole lot with the ePRO in between. Like it was the set up was the biggest part of it then after that it really didn’t, you know, come into play" (GBP02) “So this was just, almost the way of, in some cases, I didn’t have to call, because I could just see [on the ePRO] that things were going well or not. And then make a phone call based on that” (P01OV)
Subtheme 1b: Impact and value of ePRO	*Value added by ePRO*	“A lot of times we just ask patients like oh, what do you want to improve or how do you think you're going to do this, but the tool actually makes it in a concrete way and puts it down on paper or a phone or tablet or whatever so that they could visualize and see it and make it simple for them” (MSP03) “I’ve had a few patients whose health significantly deteriorated. So, one of them we redid her goals last week—or two weeks ago, because she felt [ePRO] was pressured” (GBP02) “[ePRO] forced a little bit of structure and, you know we try and help people be more specific and really set specific goals and think about, you know what outcomes they want to see” (GBP02)
Subtheme 1c: Alignment with existing workflow may influence usage behaviour	*Poor fit between ePRO and providers’ existing workflow*	“[ePRO is] not hard to use. It's just that it's an extra step. And so it isn't much time, but when you have little time, if it was, like you said, integrated into the system already, I think you'd be much more likely to use it than having to go and then sign into something else” (MSP02) “Yeah. I think though it's not easy to access it, right? Because if I'm already doing this and documenting in my chart, I'm just going to go to my previous chart note and look at what the goal was that I documented there. Versus this where it's an extra step where you have to sign into a system, right? So I think that is what makes it that might be a barrier to using it. So if it was integrated into the system that we're already using obviously it makes it easy, the appointment's going to flow easier, everything's going to happen quicker” (MSP01)
*Theme 2: Behavioural intention*		
Subtheme 2a: High behavioral intention	*Providers’ willingness to use ePRO*	“I think I would certainly be interested in trying it with more people and seeing, you know, what that looked like with different types of patients, at different stages” (GBP05) “It’s definitely easy to goal-set, I think my mindset is, you know, trying to see patients and sometimes I think you really need to set aside time, to really go over the goals and details. And so, I think what I did maybe incorrectly is try to do all of the [E-PRO] in one session, like do all of the goal-setting in one session, which isn’t realistic. And *probably looking forward*, is to do goal setting but it doesn’t all have to be in one sitting, to, to get all of the goals set, and how they’re going to achieve those goals and how we’re going to measure those goals. I think those things could be done over a long period of time, rather than trying to do it all at once” (MSP03)
Subtheme 2b: Low behavioral intention	*Providers’ lack of buy-in to ePRO*	"For me—I was—because I was one of—more the leaders of—I was sort of pushed into it quite honestly—it wasn’t a volunteer thing. And I’ll be honest, I’m not—I was—I’m a bit of a realist, a bit skeptical about some things. Not passionate about it, it was a project. I was a bit skeptical… It’s not a tool, like to be honest, that I would say I’d want to use" (GBP02) “The patient he wasn't really interested in it so yeah, there wasn't too much use of it” (MSP01)
Subtheme 2c: Behavioral intention changed over time	*Change in degree of interest in ePRO over time*	“The initial excitement happened, but then the [research] process took such a long time that the excitement disappeared” (PO2OV) “I think when you're having a conversation and not using the tool I think the conversation flows a little more easily versus when you're trying to plunk wording into a template” (MSP01)
*Theme 3: Improving usage behaviour*		
Subtheme 3a: External factors may influence usage behaviour	*Recommendations to enhance providers’ usage behaviour relating to external factors*	“I could imagine that if we were going to carry on, there would need to be that reinforcement because I think it's not just reinforcement, it's also—it also makes the providers a bit more accountable” (GBP04) “Hmm. Yeah, you’d need a lot. You’d need IT support. You’d need funding. You’d need training, definitely right?…And yeah, training not just for us, but also for the patients, like. And support for patients as well. Because the teaching usually supports us—like the provider, right. But sometimes, the patient needs that IT support too” (GBP01)
Subtheme 3b: Perceived ease of use may influence usage behaviour	*Recommendations to enhance providers’ usage behaviour relating to ePRO’s perceived ease of use/usability*	“I suppose support if there's any glitches because to me technology always has glitches, easy support, but it has to be embedded in. It has to be something I pull up, and I'm like oh, I'm using this tool to set goals with the patient, they have it in their system but I have it already in mine” (MSP04) “I think overall it was fairly easy to use. I think it's just the wording, like just how the question was set up. I think it's just maybe not the way I'm used to do it. So I remember part of it seemed redundant. So it was just kind of thinking about, thinking a little bit harder about okay, what do I put here and what do I put there and what do I put here so that it all makes sense. Hopefully that makes sense to you… I think when you're having a conversation and not using the tool I think the conversation flows a little more easily versus when you're trying to plunk wording into a template” (MSP01)

## Theme 1: perceived usefulness

This theme captured providers’ perceptions about the ePRO’s usefulness. PU was examined in relation to how well providers perceived ePRO aligned with their typical approach to care (subtheme 1a), the tool’s overall impact and value (subtheme 1b), and how well the tool fit with their existing workflows (subtheme 1c).

### Subtheme 1a: usage behaviour aligned with providers’ typical approach to care

This subtheme exemplified PU in relation to how well providers perceived the ePRO aligned with their typical approach to care. All providers reported that they wanted to help patients achieve meaningful personal goals. However, providers varied in how they pursued this aim, which influenced how they engaged with the ePRO tool (i.e. PU influencing UB; Fig. 1—arrow 1). Providers fell into one of two groups in this regard: (i) providers who viewed goal-oriented care as a self-management process and (ii) providers who viewed goal-oriented care as an ongoing collaborative process.

For some providers, goal-oriented care was a self-management process. The first group were providers who viewed goal-oriented care as a *self-management process.* For providers like MSP03 (physician) and GBP02 (nurse), a goal-oriented care approach was centred on a single visit, where providers assessed the patient and identified patient-directed goals and a care plan. The provider would supply patients with educational resources to support self-management to goal progression. Since this work typically occurred in a single visit, these providers commonly used the ePRO only once to support collaborative goal-setting and develop a goal progression plan. After the visit, the patient was responsible for initiating future discussions about the tool; these providers rarely encountered the same patient unless the patient self-identified a new concern. As a result, these providers had limited insights into how their patients were interacting with the tool, and many assumed that no news from the patient meant that the patient did not need their guidance. MSP03, who viewed the ePRO as a self-management process, reflected on his use of the ePRO:

Once we’ve collaborated and figured the goals out together. The rest, I’ve sort of left it up to the patients to dictate how they want to pursue it afterwards. Rather than me kind of bringing it up, or me, following it or measuring certain outcomes based on [the ePRO]. I primarily use it just for the goal-setting aspect (MSP03).

GBP02 was unclear whether providers were expected to follow-up with patients about the tool or whether this was the patient’s responsibility and questioned the value of a provider’s involvement beyond initial goal-setting using the tool. To support self-management, providers like GBP02 wondered whether the tool could be used independently by patients rather than with providers because patients would be hesitant to contact a provider to modify a goal. Providers like GBP02 discerned that the collaborative nature of the ePRO could make patients more dependent on providers, which contradicted their self-management efforts; this seemed to reduce the intention to use the tool.

In contrast, providers like GBP03 (dietician), POV01 (dietician), GBP06 (nurse) viewed goal-oriented care as an *ongoing collaborative process* would regularly follow-up with patients beyond the initial goal-setting visit and stressed that monitoring was a key part of their work. They argued that regular interactions with patients resulted in closer patient-provider relationships, allowed better management of a patient’s conditions, enhanced patient motivation, and could potentially reduce hospitalizations. Providers like GBP03, who regularly followed up with patients, used the ePRO to set collaborative goals with patients as well as monitor goal progression. To make ongoing check-ins with patients using the ePRO more efficient, a suggestion was to allow patients to independently input data into ePRO prior to their visit.

I did like that it flowed with the way we think in terms of our level of care and making it very patient-centred. And they’re smart goals and very achievable…I did like that it meshed with our philosophy (GBP03).

In sum, providers’ UB reflected their usual approach to care (i.e. PU influencing UB; Fig. 1—arrow 1), whether it was simply to set goals during a patient visit or set (a *patient self-management* approach to goal-oriented care), monitor and modify a patient’s goals (an *ongoing collaborative* approach to goal-oriented care).

### Subtheme 1b: impact and value of ePRO

This subtheme captured PU in regards to providers’ perceptions of the ePRO’s impact and value. While providers disagreed about the tool’s impact and value, there was a consensus that it could be a valuable resource for patients. In terms of the impact and value of ePRO for providers, providers, such as GBP05, MSP03 and P010V, believed that the ePRO had positively impacted their clinical activities (PU influencing BI and UB; Fig. 1—arrows 1 and 2). For instance, the tool had added more structure to patient visits, which in turn “helped [the provider] direct people a little bit better or support them a little bit better with goal-setting” (GBP05). A positive impact on providers’ goal-elicitation/setting processes was noted as the tool led providers to prompt patients more during goal-setting leading to more specific goals. As MSP03 explained:

Before, we would just be like, hey, go for more walks. It was really not specific at all. Now I try to say like walks are not enough. So how long? How much? How are you going to measure it? How are you going to know your results?

Providers noted that the ePRO promoted more collaboration between the provider and patient, positively impacting the patient-provider relationship. Moreover, the tool produced additional information about a patient, enabling providers to monitor a patient’s health remotely. For example, P010V described a situation where the use of the ePRO resulted in better management of a patient’s blood sugar levels:

Generally, we would check-in every three months, and because we were connecting more often, I was able to learn that her fasting blood sugar was not well controlled in that interim period, which…allowed a conversation to increase her insulin sooner rather than waiting for the three-month visit...improved overall care, in terms of blood sugar management (P010V).

Finally, providers noted that the ePRO improved communication with other healthcare providers (external factor (other providers) influencing PU; Fig. 1—arrow 3). MSP03 explained that the tool allowed them to be more specific about a patient’s goals when communicating with the interprofessional team. Some providers, however, felt that the tool simply repeated what they had already been doing.

Providers discussed their perceptions of the impact and value of ePRO for patients. The ePRO tool was seen to be valuable to increase a patient’s accountability. P03OV contended that the tool offered mutual benefit for the provider and patient, stating that the patient’s feedback was that the ePRO tool “helped her feel accountable to herself and to [P03OV] to monitor how much exercising and she was also monitoring her pain as well at the same time.” GBP04 felt this kind of accountability could also be elicited through checking in with a patient: “People like to have somebody that they can be accountable to…sometimes it’s us, maybe it could be an app.”

Providers suggested that the tool worked better for certain types of patients. They indicated that the tool worked best for patients who had realistic goals, were “driven by numbers and charts,” and most importantly, were at the readiness stage of change (i.e. willing to make changes in their health-related behaviours). GBP04 categorized these patients as “the worried well” and further elaborated that this would be “somebody that’s engaged in self-management….[and] willing to make some changes as a result of the data that we gather.” The tool had not worked well for patients not at the readiness stage or who did not have realistic goals. In cases where a patient failed to progress on their goal, the tool became a source of stress and frustration. One provider anticipated similar challenges could exist for patients who had deteriorating conditions.

### Subtheme 1c: alignment with existing workflow may influence usage behaviour

This subtheme captured PU in relation to the ePRO’s alignment with providers’ existing workflow. Providers indicated that aspects of the ePRO fit poorly with their existing workflow as the ePRO did not integrate into their existing clinical documentation system. The poor fit of the ePRO with existing workflows led to decreased use of the tool. As a result, the ePRO was perceived as “time-consuming” and created an “extra step” for providers who had to document their clinical notes twice. Due to their high caseloads, an additional step in their workflow negatively impacted a provider’s PU of ePRO and UB (PU influencing UB; Fig. 1—arrow 1).

Providers also struggled to fit goal-setting into a visit. One provider indicated that there was not enough time to use the tool to set specific goals because patients typically presented with multiple issues at each visit. As a result, they had to schedule an additional appointment with a patient to set goals on the ePRO but realized that this would not be realistic to do with all of their patients. In addition, providers also expressed dissatisfaction with the wording of some prompts within the tool (PEOU influencing PU; Fig. 1—arrow 4). MSP01 argued that the wording failed to align with providers’ preferred dialogue and could disrupt the goal-setting process as well as the patient relationship.

Finally, GBP03 and GBP06, who used the tool to check in with and monitor patients (using a collaborative care approach), believed that the tool failed to support ongoing monitoring because it did not provide notifications or reminders for providers and patients (PEOU influencing PU; Fig. 1—arrow 4). Due to the tool’s lack of ease of use, providers had to set reminders to follow-up with patients personally, and this reduced the providers’ perceptions of the tool’s usefulness. Over time, the lack of reminders led providers to discontinue using the tool.

Providers stated that enhancing the tool’s alignment with their workflow (e.g. automatically populate into their existing electronic documentation system) will enhance the tool’s PU and providers’ UB (PU influencing UB; Fig. 1—arrow 1) as providers would be “happier to adopt these sorts of tools” if they were “integrated into the EMR” (MSP03).

In sum, PU included the perceptions about how the tool improved their approach to care as well as their perceptions about the value and impact of using the tool with the specific population. PU was influenced by external factors and PEOU and influenced a providers’ BI and UB.

## Theme 2: behavioural intention

BI in this study refers to a providers’ willingness, excitement and/or motivation to use the ePRO. Providers’ BI ranged on a continuum from high (subtheme 2a) to low (subtheme 2b) and changed over time (subtheme 2c).

### Subtheme 2a: high behavioural intention

Providers with high BI believed the tool complimented or had the potential to improve their care approach or quality, with some praising the tool as “a brilliant idea.” They also believed that the tool could be a valuable self-management tool for patients with chronic diseases and described using it multiple times or expressed a desire to use it in the future (BI influencing UB; Fig. 1—arrow 5).

In addition, providers had high BI when the tool was used with patients at the readiness stage for change. For example, GBP06 explained, “[ePRO] just fit. Because it’s about goal-setting, right and patient-centered goal-setting. So, at the beginning, I actually found it quite easy, in the sense that, you know it was about setting—helping a patient set goals” (external factor (patient) influencing BI; Fig. 1—arrow 6).

### Subtheme 2b: low behavioural intention

In contrast, providers that expressed lower BI did not perceive added value from the ePRO in their work (low BI-low PU). Generally, these providers had indicated that they were not technologically adept (PEOU influencing BI; Fig. 1—arrow 7), expressed skepticism of the tool’s usefulness (BI influencing PU; Fig. 1—arrow 2) for their patient population, or they simply lacked interest in integrating a new tool in their work. Given their lack of buy-in to this tool, they preferred the ePRO to be a tool that patients would independently use, and they described a lower UB (BI influencing UB; Fig. 1—arrow 5). Lower BI was also experienced by providers who used the tool with patients that were not the right fit for the ePRO. For instance, GBP05 explained, “I ended up setting it up with—I can’t remember if it was two or three people—and following up with, I think two people. And you know, it was again, different with both people, depending on kind of how they think and how they work.” Further, GBP02 felt that the tool was an added task for patients who were already overburdened with the health care system: “When you have so many health appointments, and people and things to do and then plus you have to do this stuff, it’s just like one more thing to do” (external factor (patient) influencing BI; Fig. 1—arrow 6*).*

### Subtheme 2c: behavioural intention changed over time

BI had changed over time for some providers like P02OV and GBP01. A few providers with high BI at the midpoint experienced lower BI at the end of the trial. Various factors impacted a provider’s BI, including a lack of role clarity in ePRO and whether or not they encountered technical issues while using the tool (external factor (technology) influencing BI; Fig. 1—arrow 6). In addition, due to the design of this research study, the lengthy time from patient recruitment to trial initiation had decreased providers’ BI, and providers commented about the decrease in patients’ interest (external factor (research trial) influencing BI; Fig. 1—arrow 6). P02OV, a clinical manager, also noted that the clinical team’s BI decreased over time because they argued “we already do that” and found the tool was “extra work” (BI influencing PU; Fig. 1—arrow 2). GBP01’s BI declined after the patient was less motivated to use it (external factor (patient) influencing BI; Fig. 1—arrow 6): “I have two that have deteriorated in the last—since they’ve started on there. So, it’s really changed what their goals would be.” Together, these factors decreased BI and made providers less motivated and interested in using the tool. Noteworthy, only one provider moved from low to higher BI after realizing the tool’s value and potential impact. This provider explained that patients were managing “better than I expected” with the ePRO (GBP01) (external factor (patient) influencing BI; Fig. 1—arrow 6).

Reasons that providers with high BI discontinued using the tool included limited opportunities to use the tool because only a few patients on their caseload were participating in this study or that the patients were not at the readiness stage of change. In addition, a lack of role clarity led GBP01 to stop using the tool: “I thought I had to follow the patient. And then I was told, ‘No, no. The patient is supposed to be directing this ePRO stuff.’ So, I scaled it back.”

In sum, BI was reflected by a providers’ willingness, excitement and/or motivation to use the tool in the clinical setting, and providers had low or high BI, and for some, BI changed over time. BI was influenced by external factors and PEOU and influenced a providers’ PU of the tool and their UB.

## Theme 3: improving usage behaviour

Providers explained that their UB may be improved through external factors (subtheme 3a) and enhancing PEOU (subtheme 3b).

### Subtheme 3a: external factors may influence usage behaviour

Providers identified external factors through various recommendations for improvement (external factors influencing UB; Fig. 1—arrow 8).

Providers indicated that organizational support (e.g. leadership and IT) was important to enhance the tool’s uptake and ensure ongoing use. They explained that the use of the tool should be well-supported and actively encouraged by leadership. Based on their experience with another tool, GBP04 stated that there should be some form of follow-up or accountability for providers to use the tool or else previous tools simply “sit in a corner and gather dust.” In addition, some providers explained that they were not “tech-savvy” and would require ongoing IT support (e.g. technical assistance) for continued use (external factor (IT support) influencing UB; Fig. 1—arrow 8).

Organizational culture referred to the providers’ attitudes, thoughts, beliefs about change in an organization, including their openness for change. Regarding organizational changes in general, providers explained that the way that a new idea is introduced in a clinical setting may influence how well the idea was accepted and adopted. Adding providers’ input was considered valuable in the change management process. For instance, GBP03 reflected, “if there’s any changes coming up…our directors or managers…usually ask for feedback.” Willingness and comfort to adopt new tools and processes were believed to be influenced by the organizational culture. GBP04 explained, “it’s definitely culture based…[de-identified] is always someone who wants to get things done. We don’t move at a glacial pace, like the majority of other healthcare organizations. We’re very much, if we’ve got something that we feel is a good idea we try and back it up with some good research, and some good evidence, and the reason why we would want to do it, and then we get on with it.” Providers that worked in programs already using technology were also more willing to adopt new technologies. In addition, GBP04 explained if “the ideas are driven from the bottom up. The ideas come from the providers and I think every one of them will say that they have the flexibility and the opportunity to be creative in their programmes (GBP04),” they were more likely to be adopted (external factor (program, organization’s culture) influencing BI; Fig. 1—arrow 6*)*.

### Subtheme 3b: perceived ease of use may influence usage behaviour

Providers suggested that improving PEOU for providers and patients may promote the ePRO (PEOU influencing UB; Fig. 1—arrow 9).

Providers discussed strategies to improve their PEOU. After an initial learning curve, providers were generally comfortable with the ePRO. Though, a few technical issues persisted. Providers explained that fixing technical issues could enhance the tool’s reliability (PEOU influencing UB; Fig. 1—arrow 9). In terms of the tool’s functionality, GBP05 complained, “There were too many scales to choose from.” Providers also recommended that additional details on the graphs and reports and a free-text section for notes was needed. Providers that intended to use the tool beyond initial goal-setting recommended that it should have notifications. In particular, GBP03 discussed two types of notifications that the ePRO should provide to providers. The first was an alert when patients entered information into the tool. The second was a reminder for providers to check in with patients to signal when they needed to re-engage with the tool (PEOU influencing UB; Fig. 1—arrow 9).

To improve PEOU for patients, providers insisted that the ePRO should be easier for patients to use. For instance, installing the ePRO on a patient’s personal device rather than on a separate device. Another recommendation was to conduct joint training sessions with patients and providers. This would allow providers insight into the training content provided to patients and clarify “what the expectations are” (MSP01). Providers also suggested that more practice with the tool would be helpful.

In sum, external factors and PEOU could impact UB by increasing providers’ BI and PU.

## Discussion

This qualitative study describes the perspectives of providers regarding the ePRO’s utility and fit to support care for patients with complex care needs, usage of the ePRO, and considerations to enhance the ePRO’s implementation in primary care settings over a 9–12-month period. Leveraging the TAM as an analytic approach enabled us to identify several factors that may influence providers’ UB, including their PU (theme 1), BI (theme 2), PEOU and external factors (theme 3). As depicted in Fig. 1, these themes were interrelated. Adding to the literature on the complexity of technology adoption in clinical settings, we have identified several practical modifiable factors that may enhance the adoption of innovative tools, such as ePRO (see Additional file 2 for recommendations for ePRO). While these recommendations are specific to the ePRO tool, key themes gleaned from this study may resonate with the implementation of other digital health tools in primary care settings. To determine whether behaviour can be reliably predicted, future research could test whether integrating these recommended changes impacts UB.

Our study revealed that providers, including those on the same team and/or discipline, operationalized approaches to care differently, which impacted their perceptions of the tool’s usefulness as well as their BI and UB. This finding is consistent with previous literature that has found variability in practice style, philosophy and the use of care models like goal-oriented care, person-centred care, and self-management [43–46]. This variation within a single setting creates challenges when implementing a digital health tool that supports one approach to care. Our findings demonstrate how these variable approaches could have impacted BI and UB and have implications for technology design and implementation. From a technology design perspective, there is a need to form an in-depth understanding of different approaches to care and whether/how approaches may differ by discipline. Differences across disciplines in team-based approaches to person-centred self-management supports, which we noted in some instances, may create additional factors to manage. Following this, tools must be continually tested and refined to align to different ways that providers operationalize care. There needs to be sufficient flexibility in the technology to adapt to these variations.

Similarly, workflow fit is another critical determinant of health technology adoption [47] that may have influenced providers’ PU, BI and UB in this study. Our findings revealed that not all providers had the same goal-setting workflow, which further complicates technology design implementation. While our findings did not confirm liability concerns identified by providers in the previous ePRO trial, they confirm previous reports that a lack of integration of tools with existing clinical systems is a top barrier for adopting mHealth tools [18, 48]. Monitoring is an important component of goal-oriented care [49], which allows providers to determine whether the identified goal has been achieved/not achieved or if a modification is needed [50]. However, as Ruben & colleagues have noted, not all providers monitored or followed up with patients about their goal progression [51]; some providers disengaged after agreeing on a patient’s goals and care plan, whereas others carried out the entire process [51]. The goal-setting process may be shortened due to time constraints, training/familiarity with the goal-setting process, providers unaccustomed to this process, or constraints imposed by the program and/or practice model [44, 50]. Again, this finding suggests the need for customizable tools to accommodate different clinical approaches and workflows [47, 51, 52], which may increase PU, BI and UB.

Our findings suggest that BI varied between providers as well as within the same provider (i.e., changed over time). Furthermore, contextual factors may influence BI [53], as evidenced in growing trends of technology adoption after 2009 [54] and during the COVID-19 pandemic [55]. Given that digital solutions are being widely accepted and rapidly adopted in healthcare organizations since the pandemic (i.e. changes in environment and culture) [56], it may be possible that providers may have higher BI to use a new tool [56]. However, some with high BI stopped using the tool, suggesting that additional factors, including PEOU and external variables, influence UB. This finding suggests the need for ongoing supports and incentives/accountability to encourage the ongoing use of new tools [57].

External factors, such as appropriate training, organizational support and organizational culture, may enhance a providers’ BI or willingness to adopt a new tool in practice [47, 58]. Training may be insufficient if it focuses too heavily on the tool’s technical aspect with little regard for how the tool will impact providers’ workflow [47]. Addressing concerns (e.g. patient-provider roles, workflow fit) identified by providers and highlighting the tool’s value during training may enhance BI, PU and UB. As comfort with technology varies among providers, training may need to be individualized to each provider [58]. In addition, providers may be more willing to continue using tools if they have technical assistance, leadership support and are within an organizational culture that supports change [58–60]. Moreover, providers indicated that enhancing the tool’s usability (e.g., adding alerts and reminders, reducing redundancy) may improve the tool’s PU, BI and UB. This finding resonates with previous literature that found poor usability leads to abandonment of tools [61].

This study has some limitations. First, recruitment challenges caused a time lag between introducing the tool to providers and the trial initiation, negatively impacting providers’ BI. Expanding the inclusion criteria from Ontario-only to Canada-wide may have mitigated recruitment challenges. Second, technical glitches/errors with the tool negatively impacted providers’ BI. As technical glitches and errors are often unavoidable, it is important to set expectations for providers on the types of common glitches or issues that can present when using similar technologies. Training on practical fixes may better prepare providers to engage with technology over a longer-term. Third, as evident in Table 3, not all participants were interviewed twice due to scheduling issues and non-responsiveness from participants. As a result, we were unable to ascertain changes in these participants’ perspectives over time. In retrospect, following up with providers on a more frequent basis may have improved retention. However, it may increase the burden and reduce feasibility. Fourth, providers had a limited number of patients using the ePRO. While we intended to minimize provider burden, in retrospect, this limited opportunities for providers to meaningfully interact with the ePRO. Fifth, this study had a small sample size (n = 13) with a limited representation of physicians (n = 1). Thus, we were unable to capture whether/how discipline may influence differences in approaches to care. Future studies should explore ePRO adoption within larger samples and consider whether enablers and barriers to the use of the ePRO differ by discipline. Sixth, we have not captured comprehensive details about provider characteristics (e.g. range of age, years of experience) and the team composition at each of the three sites; this information could have further contextualized the results. Finally, there were limitations related to the design of the ePRO tool. Despite the ePRO being co-designed by end-users [62], the co-design process did not capture the variability in clinical approaches and work processes encountered in the trial settings. In future research, it may be necessary to integrate regular feedback loops as part of the trial design. In addition, taking more of an adaptive trial approach may better suit these evolving needs.

Despite these limitations, the TAM in relation to the data presented is a novel contribution to the literature. Moreover, the longitudinal nature and length of the follow-up period (i.e., 12 months) was a strength as it allowed us to identify approaches to care and workflow processes that would not have been captured during a shorter trial. In addition, interviewing providers at two different time points allowed us to capture changes in BI, which would not have been identified during a single interview. Finally, these findings have the potential to guide the implementation of other digital health interventions that are similar to the ePRO. Our research team will be applying these study findings to inform further refinements to the ePRO that will enhance providers’ adoption of the tool. For instance, to enhance greater alignment with provider workflows and UB, we intend to refine the tool’s functions to align with multiple approaches to goal-oriented patient care.

## Conclusion

This study reveals the experiences of primary care providers from three primary care settings regarding the ePRO’s utility and fit to support care for patients with complex care needs, usage of the ePRO, and considerations to enhance the ePRO’s implementation in primary care settings. Several factors that influenced UB were identified using the TAM, including the fit with providers’ practice style, workflow and organizational culture. Development and implementation of any healthcare technology require adaptive and iterative processes such that the factors that influence usage and BI meet the demanding needs of primary care providers caring for complex patients.

## Supplementary Information

**Additional file 1**. Standards for Reporting Qualitative Research checklist.

**Additional file 2**. Recommendations for the ePRO.

## Data Availability

The datasets generated and/or analysed during the current study are not publicly available due to confidentiality but are available from the corresponding author on reasonable request.

## Abbreviations

AFTHO: Association of Family Health Teams of Ontario
BI: Behavioural intention
ePRO: Electronic Patient Reported Outcome
FHT: Family Health Team
mHealth: Mobile health
PEOU: Perceived ease of use
PU: Perceived usefulness
TAM: Technology Acceptance Model
UB: Usage behaviour
