# Supplementary material for: Exploring the perspectives of primary care providers on use of the electronic Patient Reported Outcomes tool to support goal-oriented care: a qualitative study
Source: BMC Med Inform Decis Mak. 2021 Dec 29;21:366. doi: 10.1186/s12911-021-01734-0 (PMC8714873; doi:10.1186/s12911-021-01734-0)
Supplement: Supplementary file 2 — Additional file 2. Recommendations for the ePRO. [file 12911_2021_1734_MOESM2_ESM.docx]

Appendix B. Recommendation based on providers’ perspectives to enhance perceived usefulness and ease of use of the ePRO

| **Target** | **Recommendations** |
| --- | --- |
| **To enhance perceived usefulness of ePRO** | - ePRO should align with providers’ perceptions of what goal-oriented care means - Integrate ePRO with existing clinical documentation systems - Fit ePRO with time constraints of patient-provider visits - Fit ePRO with usual workflow - Emphasize the impact and value of ePRO for providers when introducing the tool - Joint ePRO training with patients and providers - Outline possible roles and expectations of providers and patients when engaging with ePRO - Patients have responsibility for ePRO to identify when provider’s need to re-engage - ePRO should not add an “extra step in providers’ workflow - ePRO training should be practical, comprehensive and aim to make providers feel comfortable with the tool (training may need to be individualized to each provider) |
| **To enhance ease of use** | - Add an alert and reminder to signal when provider needs to re-engage with ePRO - Language of ePRO should fit with clinical language - Allow ePRO to be customizable, but limit choice - Address technological errors to enhance reliability of ePRO - Anticipate providers may experience an initial learning curve |
| **External factors** | - Support and endorsement of ePRO from leadership - Ongoing, easy to access IT supports for patients and providers |
